# Dynamics and within-host interaction of *Theileria lestoquardi* and *T. ovis* among naive sheep in Oman

**DOI:** 10.1038/s41598-020-76844-2

**Published:** 2020-11-13

**Authors:** Hoyam Awad, Amal A. H. Gadalla, Milagros Postigo, Salama Al-Hamidhi, Mohammed H. Tageldin, Sini Skariah, Ali A. Sultan, Eugene H. Johnson, Brian Shiels, Arnab Pain, Joanne Thompson, Hamza A. Babiker

**Affiliations:** 1 Department of Biochemistry, College of Medicine and Health Sciences, Sultan Qaboos University, AlKhoud 123, Muscat, Oman; 2 Division of Population Medicine, School of Medicine, College of Biomedical Sciences, Cardiff University, Cardiff, UK; 3 Department of Biology, College of Science, Sultan Qaboos University, Muscat, Oman; 4 College of Agriculture and Marine Sciences, Sultan Qaboos University, Muscat, Oman; 5 Department of Microbiology and Immunology, Weill Cornell Medicine - Qatar, Cornell University, Qatar Foundation, Doha, Qatar; 6 Institute of Biodiversity, Animal Health and Comparative Medicine, College of Medical, Veterinary and Life Sciences, University of Glasgow, Glasgow, UK; 7 Biological and Environmental Science and Engineering Diversion, King Abdullah, University for Science and Technology, Thuwal, Saudi Arabia; 8 GI-CoRE, Research Center for Zoonosis Control, Hokkaido University, Sapporo, Japan; 9 Institute of Immunology and Infection Research, School of Biological Sciences, University of Edinburgh, Edinburgh, UK

**Keywords:** Microbiology, Parasitology

## Abstract

Mixed species infections of *Theileria *spp. are common in nature. Experimental and epidemiological data suggest that mixed species infections elicit cross-immunity that can modulate pathogenicity and disease burden at the population level. The present study examined within-host interactions, over a period of 13 months during natural infections with two *Theileria *spp., pathogenic (*T. lestoquardi*) and non-pathogenic (*T. ovis*), amongst a cohort of naive sheep in Oman. In the first two months after exposure to infection, a high rate of mortality was seen among sheep infected with *T. lestoquardi* alone. However, subsequently mixed-infections of *T. lestoquardi* and *T. ovis* prevailed, and no further death occurred. The overall densities of both parasite species were significantly higher as single infection vs mixed infection and the higher relative density of pathogenic *T. lestoquardi* indicated a competitive advantage over *T.* ovis in mixed infection. The density of both *species* fluctuated significantly over time, with no difference in density between the very hot (May to August) and warm season (September to April). A high degree of genotype multiplicity was seen among *T. lestoquardi* infections, which increased with rising parasite density. Our results illustrate a potential competitive interaction between the two ovine *Theileria *spp., and a substantial reduction in the risk of mortality in mixed parasite infections, indicating that *T. ovis* confers heterologous protection against lethal *T. lestoquardi* infection.

## Introduction

Malignant Ovine Theileriosis (MOT), caused by *Theileria lestoquardi,* in sheep and Tropical Theileriosis, caused by *Theileria annulata* in cattle, are widespread, tick-borne diseases in tropical and subtropical regions. As for other apicomplexan parasites, mixed infections of different *Theileria *spp. and genotypes of the same species is frequent^1–7^, due to sequential infection in areas of high transmission intensity or simultaneous infection of multiple genotypes from a single tick. The acquisition of multiple parasites is often associated with interactions that can influence the outcome of disease and the fate of each parasite within the infected host.

Within-host competitive interaction between parasites is a major evolutionary force that can shape the parasite populations and disease outcome. It may affect parasite density, transmission and virulence^8^. Understanding how the co-infecting parasites interact is central to understanding transmission dynamics and disease risk at the population level. A plausible mechanism postulated to drive parasite-parasite interaction is modulation of the host’s immune system that results in an enhanced response against other pathogens^8,9^. The influence of co-infection of one parasite on the infection outcome of another has been called “heterologous reactivity”, i.e. immunity to one pathogen reducing susceptibility to a second^9^. Consequently, the density and disease outcomes of virulent parasite strains may be less in mixed infections than in a single infections^10,11^. Studies in rodent malaria parasites have demonstrated that, in immune competent mice the avirulent *Plasmodium* strain suffered more competition than a more virulent strain, demonstrating a competitive advantage of virulent parasites in an immune-mediated interaction^11^. Similarly, an experimental study on *T. annulata*, showed higher density of the virulent clone in a mixed compared to a single infection^12^.

This study reports the first detailed longitudinal investigation of the dynamics (within-host interactions) of coinfection of two *Theileria species*, the pathogenic parasite, *T. lestoquardi*, the causative agent of MOT and the non-pathogenic, *T. ovis*, in a naïve cohort of sheep in Oman. Our previous studies in Oman revealed a high prevalence of *Theileria *spp. among livestock^13^. MOT in sheep is associated with high morbidity of 30–40% and mortality can reach up to 100% among clinical cases of indigenous breeds during seasonal epidemics^14^. However, a large proportion of sheep carry asymptomatic mixed species infections of *T. lestoquardi* and *T. ovis*^7^. The data suggest the occurrence of interactions between the pathogenic and less pathogenic species of *Theileria* and supports the hypothesis that concurrent co-infection can lead to a reduction in parasitaemia and mortality associated with the pathogenic species^9^. This premise was tested further in the current study.

## Results

### Efficiency and sensitivity of qPCR for quantification of *T. lestoquardi* and *T. ovis*

**Figure 1:**
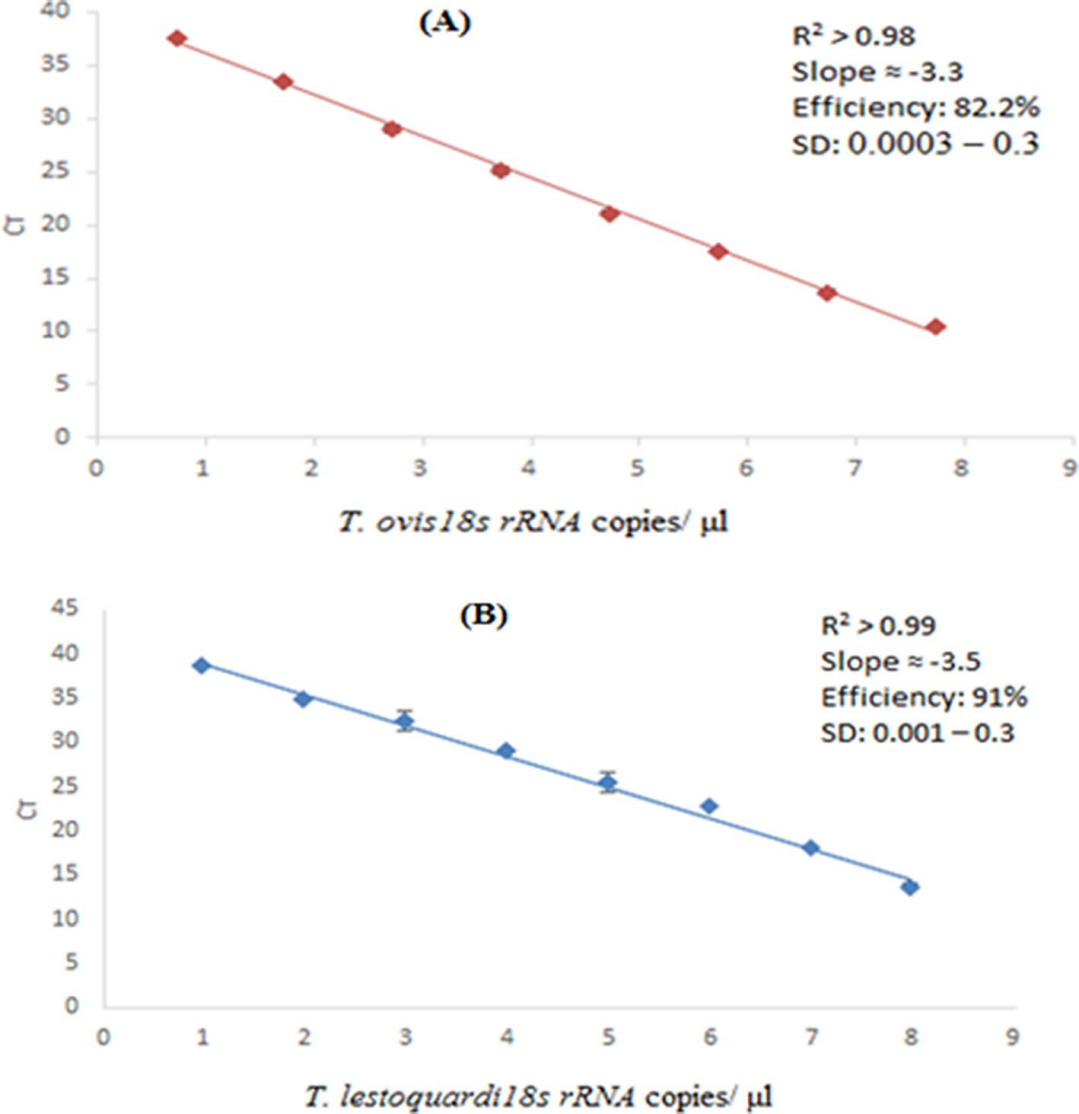
*T. ovis* (**A**) and *T. lestoquardi* (**B**) *18s rRNA* standard curves. Standard curves generated from eight points of tenfold serial dilutions of *T. lestoquardi* and *T. ovis 18s rRNA* purified pooled PCR products (amplified from pure *T. lestoquardi* culture and a natural infection with only *T. ovis* genomic DNA), with detection range of 9.26 × 10^7^–9.26 *18s rRNA* copies/ μl DNA) and 5.3 × 10^7^–5.3 *18s rRNA* copies/μl blood for *T. lestoquardi* and *T. ovis 18s rRNA*, respectively. Y axis represents the qPCR cycle threshold (C_T_). X axis represent log10 *18s rRNA* copy number.

*T. lestoquardi* and *T. ovis*-specific qPCR assays were developed to estimate parasite density and investigate within-host interaction between the two ovine *Theileria *spp. endemic in Oman. Initially, assay indices considered indicative of a well-optimized qPCR were assessed. This included the linearity of data (*R*^2^ > 0.98), an efficiency (E) value within the range of 80–100% and consistency of Cq values across replicates. The qPCR amplification efficiency was 91% and 82% for *T. lestoquardi and T. ovis 18s rRNA* genes, respectively. The inter-assay variability between standard curves was 2% and 1% for the *T. lestoquardi 18s rRNA* and *T. ovis 18s rRNA* assays, respectively, while the correlation between log_10_ *18s rRNA* copies and Cq values was significant for both species (*T. lestoquardi* adjusted R^2^ > 0.99 for all PCRs with *P* < 0.001; *T. ovis* adjusted R^2^ > 0.98 for all PCRs with *P* < 0.001). A high consistency of Cq values across replicates was seen, the standard deviation^15^ ranged between 0.001–0.3 and 0.0003–0.3 for *T. lestoquardi* and *T. ovis 18s rRNA* qPCR, respectively. The limit of detection^3^ was 9.26 log_10_ and 5.3 log_10_ *T. lestoquardi* and *T. ovis 18s rRNA* copies/μl blood, respectively (Fig. 1).

### Analysis of *T. lestoquardi* and *T. ovis* infected sheep

Fifty sheep (North Oman breed) were transferred to an endemic farm in April 2016. There was minimal difference in the mean age of the examined sheep (31–32 weeks) and all sheep were kept exclusively indoors, in a small open enclosure attached to the farmer’s residence, with similar expected levels of exposure to ticks, *Hyalomma anatolicum*. At the early stages of the study, May and June 2016, 3 and 4 sheep died of suspected MOT, respectively. *Theileria* species identification and density were determined in blood samples collected from 43 sheep between May 2016 and May 2017 (13 times points per sheep). No parasites were seen when examined by Giemsa stained blood film and microscopy for the majority of the samples. However, occasionally animals showed parasites detected by microscopy during the follow up period. Interestingly, a very high parasite density (piroplasm stage) was seen in one dead animal examined few hours prior to death. Therefore, *Theileria ssp* were detected and infection levels assessed primarily by qPCR.

Infection was defined as single species, when the initial infection in May 2016 was diagnosed with either *T. lestoquardi* or *T. ovis* alone and continued in consecutive months till a mixed species infection (*T. lestoquardi* plus *T. ovis*) was seen. As the outcome of interaction between the two species can be influenced by the immune response^9^, the appearance of a single species following the detection of mixed species was not defined as single infection.

*Theileria *spp. could be detected in 485 (91%) of the 533 (95%) blood samples, collected during the study period; *T. lestoquardi* alone was detected in 125 (23%) and *T. ovis* alone was detected in 4 (1%) of the samples. Thus, a mixture of both species was detected in the majority of blood samples**.** The lower prevalence of single *T. ovis* differs from an earlier survey in Oman, (April and August 2014) when single *T. ovis* infection was more dominant than single *T. lestoquardi*^6^. The lower prevalence of single *T. ovis* among the present cohort is unlikely to be due to technical deficiency generating false negative results, as the limit of detection of the *T. ovis* specific qPCR assay was two-fold higher than that of *T. lestoquardi*.

### Pattern of Theileria species infection and mortality

**Figure 2:**
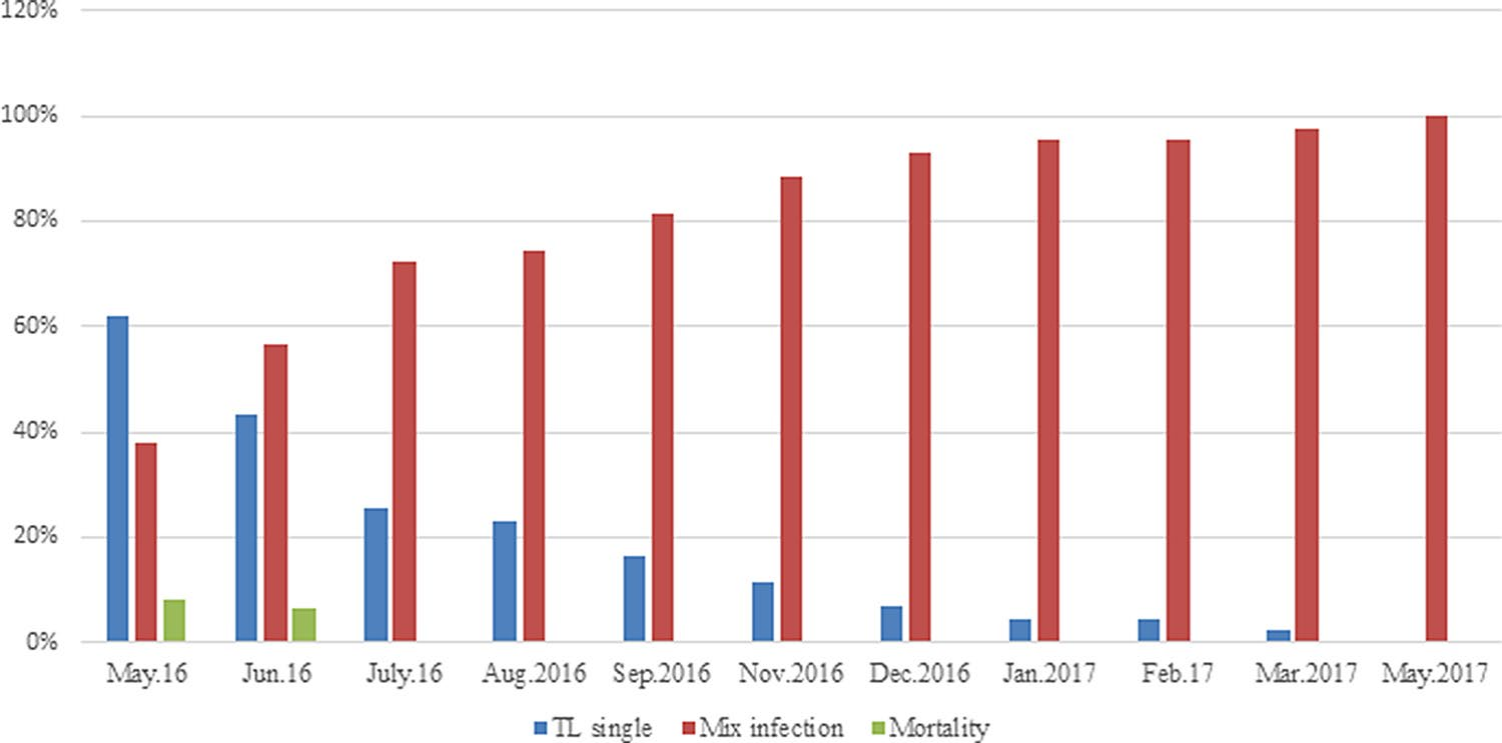
Proportions of *T. lestoquardi* and *T. ovis* infection and mortality in a cohort of sheep (n = 50), over a period of 13 months (May 2016 to May 2017) in a farm in Sharqiyah region, Oman. 7 animals died, 4 (8%) in May 2016 and 3 (7%) in June 2016, while no deaths occurred after June 2016.

Figure 2 shows the pattern of *T. lestoquardi* and *T. ovis* infection and mortality in a cohort of sheep (n = 50), over a period of 13 months (May 2016 to May 2017). At the start of the study (April 2016) 50 apparently healthy and uninfected sheep were transferred to a known area of *Theileria* transmission. In May 2016 (second sampling point) all animals had become infected; 62% had single *T. lestoquardi* infection, 38% had mixed infection (*T. lestoquardi* plus *T. ovis*), and no single *T. ovis* infections were detected. At this point, 3 sheep died, and an additional 4 animals died in June 2016. In subsequent sampling of the remaining 43 animals, single *T. lestoquardi* infections decreased dramatically reaching 2% by March 2017 and zero in May 2017. In contrast, mixed infections increased from 38% in May 2016 to 100% in May 2017 (Fig. 2, Supplementary Table 1). Disease symptoms (enlarged superficial lymph nodes, high fever and anorexia) consistent with theileriosis were observed in the first two months (May and June 2016). During this period, there were cases of mortality (7 out of 50 animals) and a high rate of single *T. lestoquardi* infection (50.8% of cohort). No other pathogens or clinical signs associated with ovine diseases known to occur in the study region were recorded. Parasite infection was detected by PCR and examination of the 7 dead showed *T. lestoquardi* schizonts and piroplasms in a few animals**.** Investigation of the 7 dead animals revealed enlarged superficial lymph nodes, while necropsy of one animal also showed an enlarged liver and spleen. Potential increase in overt clinical signs assessed in surviving single infected animals may have occurred earlier in the infection period, prior to death, between the two time points, but were not taken. All but one of the animals that died, were infected by *T. lestoquardi* alone, and mortality was significantly associated with single *T. lestoquardi* infection (Fisher’s exact test, *P* < 0.001).

The parasite population seen in each of the dead animals, prior to death, was comprised of a different, distinct combination of multiple genotypes, as identified by the pattern of alleles generated by five neutral microsatellite markers (Fig. 2, Supplementary Table 1). Thus a specific virulent population or over representation of certain genotypes could not be associated with death in these animals.

### Dynamics and interaction between *T. lestoquardi* and *T. ovis*: parasite infection

**Figure 3:**
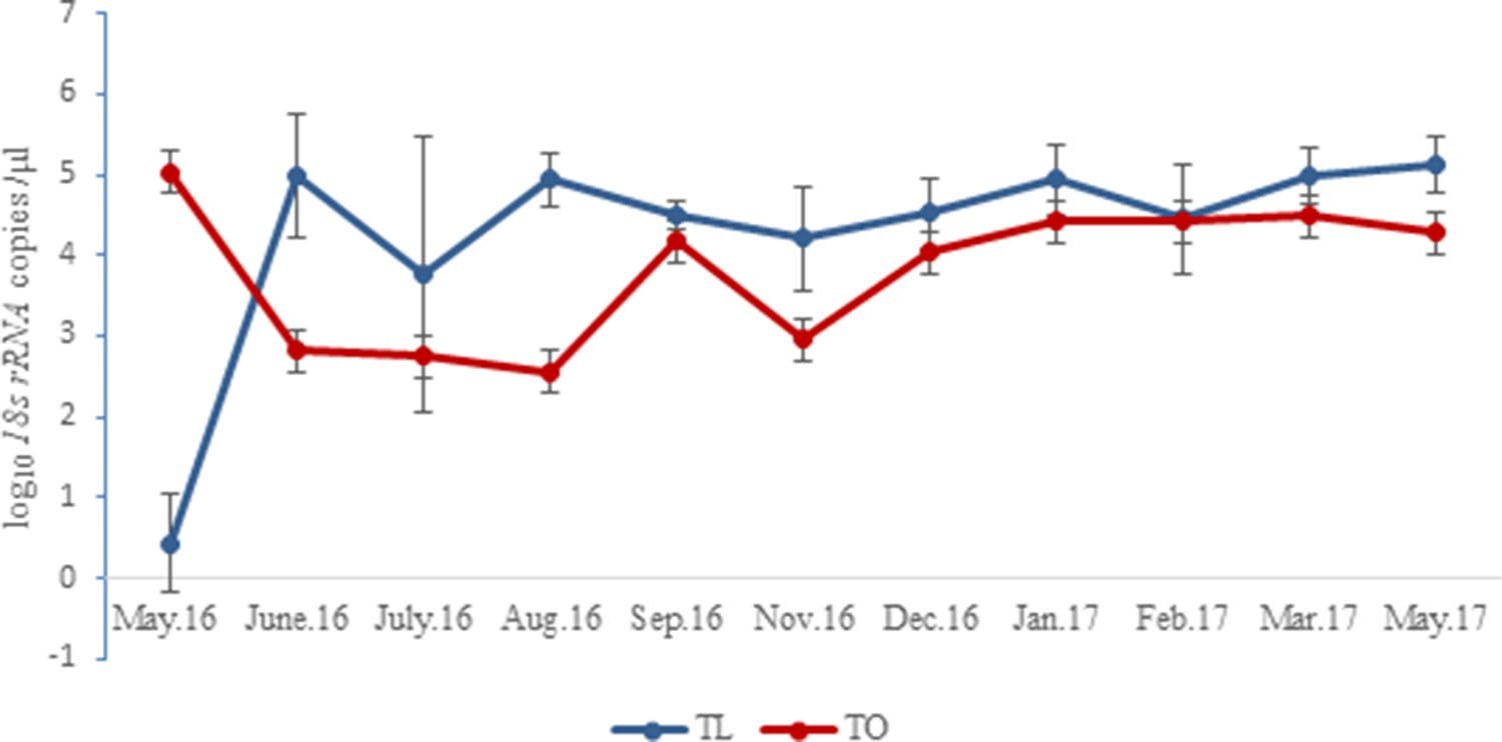
*T. lestoquardi* density (blue) and *T. ovis* density within mixed species infection. *T. lestoquardi* density was higher than *T. ovis* density throughout study period, except in May 2016. Error bars represent 95%CI.

Following the acute phase of MOT, 43 (86%) sheep maintained chronic asymptomatic *Theileria *spp. infection throughout the 13 months of the study period, most often as a mixed infection detectable by qPCR. Figure 3 shows the persistence and temporal dynamics of *T. lestoquardi* and *T. ovis* detected among sheep with mixed species infection, using *18s rRNA gene* copy number estimated by qPCR as a proxy measurement of parasite density.

The estimated density of *T. lestoquardi* fluctuated significantly during the study period (log likelihood: − 636.91, df: 10, *x*^*2*^: 51.582, *P* < 0.001), ranging between 2.8 log_10_ *18s rRNA* copies/μl (95% CI 1.3–4.3 log_10_) in May 2016 to 5.1 log_10_ *18s rRNA* copies /μl blood (95% CI 4.9–5.4 log_10_) in May 2017. A similar pattern was seen for *T. ovis* (Log likelihood: − 571.9, df: 10 *x*^*2*^: 39.77, *P* < 0.001) (Fig. 3).

When the study period was divided between the two distinct climatic seasons of the region; very hot (May to August) and warm (Sept to April), no seasonal linked changes in density were seen for either parasite; *T. lestoquardi* (log likelihood: − 661.58, df: 1, *x*^*2*^: 2.254, *P* = 0.133) and *T. ovis* (log likelihood: − 591.83, df: 1, *x*^*2*^: 0.086, *P* = 0.769). The estimated average *T. lestoquardi* density was slightly higher (4.7 log_10_ *18s rRNA* copies/μl blood, 95% CI 4.5–4.9 log_10_) in the warm season compared to the very hot season (4.5 log_10_ *18s rRNA* copies/μl blood, 95% CI 4.0–4.9 log_10_). Conversely, for *T. ovis* the estimated average density was slightly lower (3.7 log_10_ *18s rRNA* copies/μl blood, CI 3.4–4.1 log_10_) in the warm season compared to the hot season (4.5 log_10_ *18s rRNA* copies/μl blood, CI 4.1–4.9 log_10_).

### Interaction between *T. lestoquardi* and *T. ovis* in mixed infection

The detection of mixed infections of *T. lestoquardi and T. ovis* increased from 36% in May 2016 to over 80% by Sep 2016 among the examined sheep and all animals with mixed infection, as well as the few persisting single *T. lestoquardi* infections, remained asymptomatic and apparently healthy (Fig. 2). We therefore tested whether the interaction between *T. lestoquardi* and *T. ovis* among these animals was associated with modulation of the load of either parasite.

**Figure 4:**
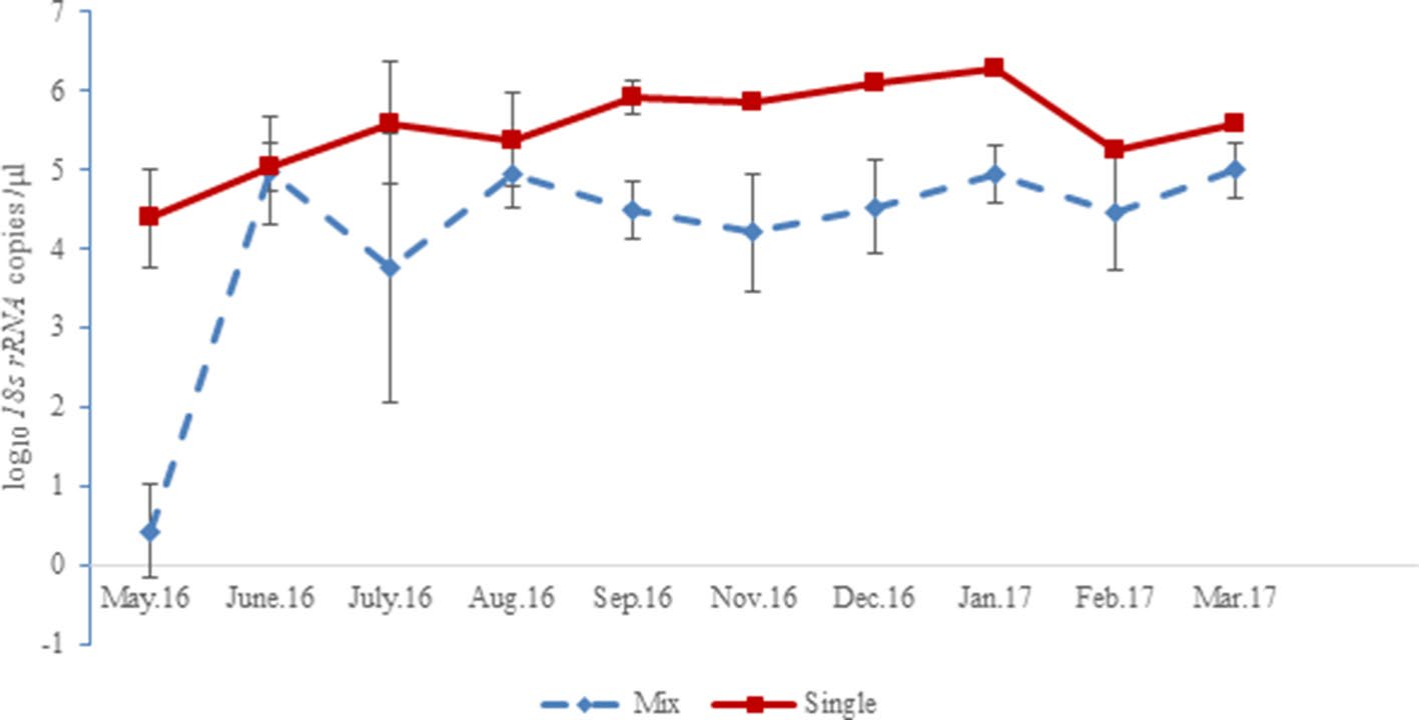
Estimated density in single (*T. lestoquardi* alone) and mixed infection (*T. lestoquardi* plus *T. ovis*) of sheep. There was a significant increase in *T. lestoquardi* density in single infection over time (Log likelihood: − 630.08, dF: 9, *x*^*2*^: 13.658, *P* < 0.001). Error bars represent 95% CI.

The estimated *T. lestoquardi* density was higher (Log likelihood: − 662.7, df: 1, *x*^*2*^: 0.014, *P*: 0.90) in single infection (5.2 log_10_ *18s rRNA* copies/μl blood, 95% CI 4.9–5.5 log_10_), compared to mixed infection (4.5 log_10_ *18s rRNA* copies/μl blood, 95% CI 4.2–4.8 log_10_). In addition, differences in *T. lestoquardi* densities in single vs mixed infection was more pronounced, generally, over time, when time as a variable was added to the model (Log likelihood: − 630.08, df: 9, *x*^*2*^: 13.658, *P* < 0.001). For example, the mean *T. lestoquardi* density in single infection in May 2016 and in March 2017 (4.4 and 5.9 log_10_ *18s rRNA* copies /μl blood, 95% CI: 3.6–5.2 log_10_) were clearly higher than the density of mixed infections (0.4 and 5.1 log_10_ *18s rRNA* copies /μl blood, 95% CI: − 0.5–1.4 log_10_) (Fig. 4).

Where detected (4 time points), single infection *T. ovis* density was significantly greater compared to mixed infection with *T.* *lestoqurdi* for the same time points (Log likelihood: − 426.44, dF: 10, *x*^*2*^: 291.07, *P* < 0.001). However, in mixed infections, *T. lestoquardi* was detected at higher density than *T. ovis* in the majority of the examined time points (Fig. 3).

### Multiplicity of *T. lestoquardi* genotypes

Previous analyses generated evidence of a high rate of multiplicity of *T. lestoquardi* genotypes in infected sheep in Oman^7^. The multiplicity of infection detected by five polymorphic microsatellites among animals in the current study ranged between 80.8% and 100%. In the first month of the study, 38 (80.8%) out of the 47 sheep examined were infected with multiple genotypes of *T. lestoquardi*. At this time, the estimated minimum mean number of genotypes per sheep among the cohort was 2.1. Genotype multiplicity and therefore the mean number of clones/genotypes (MNC) of *T. lestoquardi* per infected sheep increased significantly over time (Log likelihood: − 460.88, df: 10, *x*^*2*^: 48.538, *P* < 0.001), from 2.1 (95% CI 1.8–2.4) in May 2016 to reach 2.9 (2.6–3.1) and 3.0 (95% CI 2.7–3.2) in September 2016 and March 2017, respectively. This suggests the occurrence of superinfection over time via successive infestations of multiple infected ticks.

**Figure 5:**
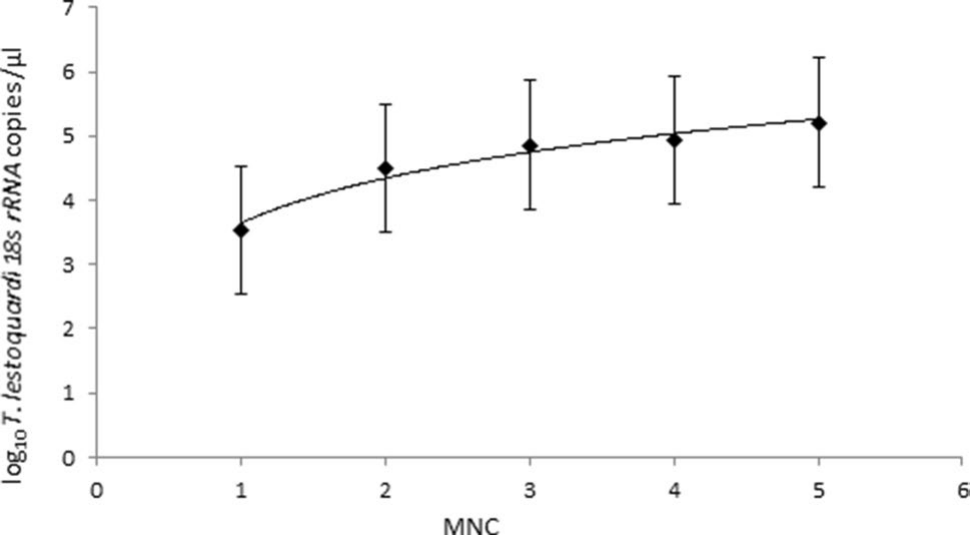
Effect of multiplicity of infection on *T. lestoquardi* density: There is a positive association between the Log_10_ *T. lestoquardi* density and the estimated mean number of clones. Black squares represent the mean values of Log_10_ *T. lestoquardi* density at each MNC. Error pars represent 95% CI of the mean value.

*T. lestoquardi* density positively associated with MNC (Log likelihood = − 615.46, df: 1, χ^2^: 20.55, *P* < 0.001): *T. lestoquardi* specific 18rRNA copies/μl DNA, was 3.54 (95% CI 2.60–4.48), 4.49 (95% CI 4.26–4.72), 4.86 (95% CI 4.70–5.01), 4.93 (95% 4.70–5.16) and 5.21 (95% CI 4.43–5.99), when the MNC was 1, 2, 3, 4 and 5 clones/genotypes respectively (Fig. 5). There was no difference in MNC between single *T. lestoquardi* 2.64 (95% CI 2.45–2.84) and mixed infections (*T. lestoquardi *and *T. ovis*) 2.64 (95% CI 2.55–2.74) (Log likelihood = − 463.42, df: 1, χ2: 1.048, *P* = 0.306). This suggests similar levels of diversity of *T. lestoquardi* in animals with single and mixed species infection.

## Discussion

This natural challenge study revealed the long-lasting persistence (over a period of 13 months) of *T. lestoquardi* and *T. ovis* parasites as asymptomatic mixed species infections in naïve sheep in Oman, and provided evidence of competitive interactions between these two parasites. The more pathogenic *T. lestoquardi* was associated with higher density and mortality when present as a single infection relative to mixed infection with the non-pathogenic *T. ovis*. Thus, co-infection with the two parasites species correlated with reduced mortality of MOT in sheep*,* supporting the hypothesis of heterologous protection proposed for other combinations of *Theileria* spp. mixed infection ^9^.

*T. lestoquardi* was associated with higher density when present as a single infection than where it was mixed with the non-pathogenic *T. ovis* (Figs. 3 and 4). A similar pattern was seen with *T. ovis* in mixed infection compared to the few occasions when it existed as single infection (4 time points), demonstrating within host interaction between the two parasites. This is evident by the fact that density of both species in mixed infection fluctuated around a threshold over the study period, while the animals were apparently healthy, with a tendency to return to an equilibrium value (Fig. 3), indicating a cross-species mechanism of regulation*.* In mixed parasite infection, within-host interactions resulting in lower virulence can occur through several mechanisms, including heterologous innate immunity^9^. Within-host selection may also favour traits that allow the parasite to avoid immune responses and survive at low densities^16^. A low degree of serological cross-immune reactivity has been detected between *T. ovis* and *T. lestoquardi* together with the relatively long duration of a detectable antibody response^17^, but it is unclear whether this provides cross protection against disease. There is evidence that immunity to *T. lestoquardi* in sheep involves T cell mediated responses^18^ against the macroschizont infected leukocyte and similar immune responses have been reported for *T. annulata* and *T. parva*^19^. Genetic and antigenic similarities are evident for *T. lestoquardi* and *T*. *annulata*, and cross-immunity between the two species has been demonstrated^17,20^. However the disease produced by these parasites are distinct from *T. ovis*, where amplification of the macroschizont infected leukocyte does not occur to any known extent, and replication is more apparent within erythrocytes similar to *T. orientalis*^21^. Thus, it is debatable whether the reduced parasite density in mixed infections found in this study relate to an acquired immune response: non-specific mechanisms linked to innate immunity and/or growth inhibition should be considered for further investigation.

However, *T. lestoquardi* was associated with higher density when present as a mixed infection with the non-pathogenic *T. ovis*. This suggests a prevailing competitive advantage of *T. lestoquardi* over the non-pathogenic, *T. ovis*. This is evident by the relatively lower density of *T. lestoquardi* in mixed infection compared to single infection. The relative density pattern in mixed species infection could be, explained by a competitive advantage of the pathogenic parasite (*T. lestoquardi*) in an immune-selection (potentially via innate immunity) environment, where it is more likely to overcome nonspecific immune responses, and build up higher densities^22^. However, the suppressive environment of mixed infection on *T. ovis*, has not been tested, as few animals in cohost displayed single *T. ovis*. Nonetheless, on the few occasions when single *T. ovis* infection (n = 4) appeared during the follow up period, as in these few samples *T. ovis* density was always relatively higher compared to mixed infection (Samples, 3981, 3986, 3988 and 3995; Supplementary Table 1). The above findings may be consistent with experimental studies using genetically-distinct strains of the rodent malaria parasite, *Plasmodium chabaudi,* in mice, where the more virulent parasite competitively suppresses a less virulent one in mixed infection^11^. Similarly, observational data from multiple *Plasmodium* parasites also suggest the existence of competitive suppression^23^. The dynamics of multispecies *Plasmodium* infection in asymptomatic carriers, under intense transmission provide evidence for a density-dependent regulation that transcends species as well as genotypes^23^. Whether this form of interspecies competition operates in ovine Theileria infection, or one species is simply more resistant to detrimental conditions than the other requires further study.

The delayed appearance of *T. ovis* among the examined cohort (Fig. 3) was not expected from previous reports on species prevalence, but may be attributed to differences in the kinetics of proliferation of the two parasites. *T. ovis* parasites are not detected in the blood until the large macroschizont stages mature and merozoites invade erythrocytes, whereas for *T. lestoquardi,* asexual multiplication occurs within leukocytes within the first week of infection with parasites entering red blood cells in week two^24^. Nevertheless, despite the possibility that *T. lestoquardi* may competitively suppress *T. ovis* in mixed infections, *T. ovis* is widespread in Oman^6^, indicative of a high transmission capacity. A high level of *T. ovis* transmission is also reflected in the increased rate of mixed infection over time seen our study. The two parasites are transmitted by *Hyalomma anatolicum*, which was the main tick species identified on the examined animals and is widely distributed in Oman^6^.

A high level of genetic diversity and genotype multiplicity of *T. lestoquardi* was detected in samples from the infected cohort of sheep. Almost all infected animals displayed a novel (or combination of) genotype (defined by unique combinations of alleles detected by the examined microsatellites), including strains extracted from dead animals. This is consistent with the high genetic diversity and multiplicity of infection reported for *T. lestoquardi* populations in Oman^7^ and Sudan^25^. Moreover, the observed increase in *T. lestoquardi* multiplicity of infection over time can be attributed to super-infection and the consecutive acquisition of novel genotypes combined with delayed clearance of initial infecting parasites. The study site is known for high abundance and infestation of *Hyalomma anatolicum*^6^, and this is predicted to promote cross-mating, genetic recombination and generation of novel parasite genotypes.

Multiple genotypes were common, among a single and mixed *T. lestoquardi* infection, while total parasitemia exhibited fluctuation around a limit (Fig. 4), suggesting a similar mechanism for regulation of the dynamics of species- and/or genotypes. The similar level of *T. lestoquardi* diversity detected in animals with single and mixed species infection suggests that both forms of infection are equally susceptible to establishment of distinct multi genotype populations. The frequent cross-mating, and random re-assortment of alleles on different gens, generate novel genotypes in infected ticks which can readily infect sheep. Following the acute phase of infection, however, protective immunity acquired by the animals does not shield from reinfection, a scenario similar to that reported for *Plasmodium*^26^. However, over time chronic multi-clonal (mixed genotypes) super-infection is considered to promote a status of ‘premunition’ with elevated protection against a re-occurrence of disease^26^, leading to higher genotype complexity in these individuals. Such findings are in agreement with the significant increase in level of *T. lestoquardi* genotypic diversity in animals, with both mixed and single infection, without mortality, over time among the examined cohort in this study.

In conclusion, the limited data from this study on the outcome of ovine theileriosis in Oman indicates that single *T. lestoquardi* infection is associated with high mortality, and that mixed species infections are associated with *T. lestoquardi* density regulation, indicative of within-host interaction, and lower mortality. A protective effect of mixed species infection (*T. lestoquardi* + *T.ovis*) against severe MOT, are in line with the results of a field epidemiological study in indigenous African calves that demonstrated an 89% reduction in mortality due to *T. parva* infection (East Coast Fever) in the presence of less pathogenic *Theileria *spp. (*T. mutans* and *T. velifera*)^9^. Further studies of mixed infections of *T. lestoquardi* and *T. ovis* are required to validate whether heterologous protection significantly reduces the impact of morbidity and mortality of MOT. In addition, the role of mixed infection in the epidemiology of ovine theileriosis should be investigated to determine whether infection of the non-pathogenic *T. ovis* can be utilised as a predictor, and control strategy, for improved clinical outcome.

## Material and methods

### Study area and subjects

This study was conducted in Sharqiyah province, eastern Oman, where our previous baseline studies identified high *Theileria *spp. (*T. annulata*, *T. lestoquardi* and *T. ovis*) infection rates among cattle (T. annulata only, 72%), sheep (*T. lestoquardi* and *T. ovis*, 50%) and goats (*T. lestoquardi* and *T. ovis*, 9.1%)^6^. A cohort of 50 new-born (3-months-old) local Omani sheep (North Oman breed; 35 males and 15 female) was raised on a tick-free farm at Sultan Qaboos University and then transferred to a farm endemic for theileriosis in Sharqiyah province, in April 2016. The North Oman sheep breed is indigenous to Oman and is considered more tolerant of *Theileriosis* than other imported breeds^14^. Animals acquired *Theileria* infection naturally and clinical data and blood samples for DNA extraction were collected every month over a period of one year (May 2016 to May 2017).

### Ethical statement

The project adhered to the guidelines and ethics code of animal welfare, approved by the Animal Care and Use Committee of Sultan Qaboos University, Oman. The blood samples were collected under supervision of a veterinarian, a non-invasive method was used to restrain animals, and blood samples collected by experienced technical staff of the Ministry of Agriculture and Fisheries, Oman. The blood samples collected solely for the purpose of this study and have not been used or will be used for another purpose. The volume of blood collected from each animal, at each time point, is compliance with the guidance of the Animal Care and Use Committee of Sultan Qaboos University, Oman. Informed consent was taken from the farm owner before drawing blood from animals.

This research was also performed in accordance with the relevant guidelines and regulations of ethical and animal welfare of the Ministry of Agriculture and Fisheries, Oman.

### Detection and quantification of *T. lestoquardi* and *T. ovis*

The density of *T. lestoquardi* and *T. ovis* parasites in whole blood was quantified using a real-time polymerase chain reaction (qPCR) assay. Primers and probes targeting *18S rRNA gene* of *T. lestoquardi* and *T. ovis* were designed (Table 1) using available GenBank sequences for *T. lestoquardi* and *T. ovis*, as described^6^. The specificity of *T. lestoquardi and T. ovis 18S rRNA gene* primers was tested against pure *T. lestoquardi*, *T. ovis* and *T. annulata* DNA samples. The *T. lestoquardi 18S rRNA* primer amplified *T. lestoquardi* DNA samples while no amplification was detected with *T. ovis* and *T. annulata* DNA. Similarly, *T. ovis 18S rRNA* gene primers amplified *T. ovis* DNA but not *T. lestoquardi* or *T. annulata* DNA.

**Table 1:** *T. lestoquardi 18s rRNA* and *T. ovis 18s rRNA* specific qPCR assays.

Name	Sequence	Location
*TL18s rRNA*-FW	GGGTCTGTGCATGTGGCTTT	476–495
*TL18s rRNA*-RV	AAATTAGAGTGCTCAAAGCAGG	536–557
*TL18s rRNA*-Pr	6FAM-TCGGACGGAGTTTCTTTGTCTGAATGTTT-TAMRA	498–557
*TO18*s*rRNA*-FW	CTTGCGGTGTACGGTGATTC	40–59
*TO18s rRNA*-RV	CAGCTTTGGACGGTAGGGTA	119–138
*TO18s rRNA*-Pr	6FAM- TGCGAATCGCGTCTTCGGATGCG—TAMRA	70–92

Sequences for forward primers (FW), reverse primers (RV) and TagMan probe (Pr) are shown with their location, based on genbank sequence.

For assay optimization, genomic DNA was extracted from cultured *T. lestoquardi* and a natural infection with *T. ovis. The 18S rRNA* genes were amplified using primers described in Table 1, and purified using Wizard SV Gel and a PCR clean-up system (Promega). Purified PCR product concentration was measured by Spectrophotometry (Nanodrop), and the copy numbers of *18S rRNA* genes for both species were calculated using the following equation. DNA concentration (ng) × 6.022 × 10^23^/ length of DNA product (bp) × 1 × 10^9^ × 650^27^. The estimated *18S rRNA* gene copies were 9.26 × 10^10^ /μl DNA and 5.3 × 10^10^ /μl DNA for *T. lestoquardi* and *T. ovis*, respectively. Eight points of tenfold serial dilutions (TL: 9.26 × 10^7^–9.26 *18s rRNA* gene copy/μl DNA: TO: 5.3 × 10^7^–5.3 *18s rRNA* gene copy/μl DNA) were used to generate standard curves.

DNA was extracted from 200 µl blood of the examined/sampled sheep, using the QIAamp DNA mini kit (Qiagen). Real-time quantitative PCR was performed in Micro Amp Fast optical 96-well reaction plates using the ABI PRISM 7500 Sequence Detection System (Applied Biosystems) with 3 μl of the extracted DNA in 20 μl PCR mix containing 10 μl of 2X TaqMan Universal PCR Master Mix, 0.6 μl of 10 µM of each primer and 0.2 μl of 10 µM probe. The reaction temperature profile was 50 °C/2 min, 95 °C/10 min, 45 cycles of 95 °C/15 s and 60 °C/1 min.

### Genotyping of *T. lestoquardi*

Five polymorphic *T. lestoquardi* specific microsatellites, TL_MS05, TL_MS07, TL_MS13, TL_MS280 and TL_MS281 were used to genotype samples, as described previously^7^. Multiplicity of infection was then determined as any infection with more than one allele at the examined loci, and the minimum number of clone/genotypes (MNC) defined by the maximum number of alleles at any locus per infection.

### Statistical analysis

Generalized linear mixed models (GLMMs) were used to estimate; (i) changes in prevalence and densities of *T. lestoquardi* and *T. ovis* over time^4^, (ii) temporal change in *T. lestoquardi*/ *T. ovis* density among single infections (*T. lestoquardi* alone or *T. ovis* alone) and mixed species infections (*T. lestoquardi* plus *T. ovis*) and (iii) association of *T. lestoquardi* MOI and *T. lestoquardi* density in single and mixed species infections.

### Response variables and model fitting strategy

A variety of distributions (Binomial, Negative binomial, Poisson and Gaussian, gaussian zero-inflation) were tested to describe the distribution of *T. lestoquardi* and *T. ovis* density between animal hosts. A Gaussian and Gaussian zero inflation distribution gave the most parsimonious fit for *T. lestoquardi* count data (Log Likihood: − 662.70, *x*^*2*^:178.220, *P* < 2e-16) and for *T. ovis* data (Log Likihood: − 591.87, x2: 418.4, *P* < 2.2e−16), respectively. Zero inflation parameters considered the fact that many samples were negative for *T. ovis* at different time points, especially during the first months of the experiment. The *T. lestoquardi* and *T. ovis* density was allowed to vary between hosts by including them as a random effect in the models. This facilitates detection of changes in density over time, as there is substantial inter-host variability. Models were built using the forward stepwise selection method and the Log likelihood ratio test (LRT) was used to determine the most parsimonious fit. When more than one model significantly fitted the data, the one with the lowest Akaike Information Criterion (AIC) was selected^28^.

### Explanatory variables

Fixed effect variables were (i) month of follow-up^4^, complexity of infection, mixed infection (*T. lestoquardi* plus *T. ovis*) or single infection (*T. lestoquardi* alone or *T. ovis* alone) at any time point during the study and (iii) multiplicity of infection of *T. lestoquardi* and the minimum number of clones, defined as the maximum number of alleles across the examined five microsatellites per infection.

## Supplementary information

Supplementary Table 1.

Supplementary Legend.

## References

[1] Mayxay M, Pukrittayakamee S, Newton PN, White NJ (2004). Mixed-species malaria infections in humans. Trends Parasitol..

[2] Iseki H, Alhassan A, Ohta N, Thekisoe OM, Yokoyama N, Inoue N, Nambota A, Yasuda J, Igarashi I (2007). Development of a multiplex loop-mediated isothermal amplification (mLAMP) method for the simultaneous detection of bovine Babesia parasites. J. Microbiol. Methods.

[3] Jalali SM, Jolodar A, Rasooli A, Darabifard A (2016). Detection of *Theileria lestoquardi* cross infection in cattle with clinical theileriosis in Iran. Acta Parasitol..

[4] Zaeemi M, Haddadzadeh H, Khazraiinia P, Kazemi B, Bandehpour M (2011). Identification of different Theileria species (*Theileria lestoquardi*, *Theileria ovis*, and *Theileria annulata*) in naturally infected sheep using nested PCR–RFLP. Parasitol. Res..

[5] Györke A, Pop L, Cozma V (2013). Prevalence and distribution of Eimeria species in broiler chicken farms of different capacities. Parasite.

[6] Al-Fahdi A, Alqamashoui B, Al-Hamidhi S, Kose O, Tageldin MH, Bobade P, Johnson EH, Hussain A-R, Karagenc T, Tait A (2017). Molecular surveillance of Theileria parasites of livestock in Oman. Ticks Tick-borne Dis..

[7] Al-Hamidhi S, Weir W, Kinnaird J, Tageledin M, Beja-Pereira A, Morrison I, Thompson J, Tait A, Shiels B, Babiker HA (2016). *Theileria lestoquardi* displays reduced genetic diversity relative to sympatric *Theileria annulata* in Oman. Infect. Genet. Evol..

[8] Vaumourin E, Vourch G, Gasqui P, Vayssier-Taussat M (2015). The importance of multiparasitism: examining the consequences of co-infections for human and animal health. Parasites Vectors.

[9] Woolhouse ME, Thumbi SM, Jennings A, Chase-Topping M, Callaby R, Kiara H, Oosthuizen MC, Mbole-Kariuki MN, Conradie I, Handel IG (2015). Co-infections determine patterns of mortality in a population exposed to parasite infection. Sci. Adv..

[10] Mackinnon MJ, Read AF (1999). Genetic relationships between parasite virulence and transmission in the rodent malaria Plasmodium chabaudi. Evolution.

[11] de Roode JC, Pansini R, Cheesman SJ, Helinski ME, Huijben S, Wargo AR, Bell AS, Chan BH, Walliker D, Read AF (2005). Virulence and competitive ability in genetically diverse malaria infections. Proc. Natl. Acad. Sci..

[12] Taylor LH, Welburn SC, Woolhouse ME (2002). Theileria annulata: virulence and transmission from single and mixed clone infections in cattle. Exp. Parasitol..

[13] Al-Fahdi, A. *Molecular Identification and Phylogenetic Studies of Theileria Parasite in Oman*. 2015, Sultan Qaboos university.

[14] Tageldin MH, Fadiya AA-K, Sabra AA-Y, Ismaily SIA-I (2005). Theileriosis in sheep and goats in the Sultanate of Oman. Trop. Anim. Health Prod..

[15] Menegon M, Talha AA, Severini C, Elbushra SM, Mohamedani AA, Malik EM, Mohamed TA, Wernsdorfer WH, Majori G, Nour BYM (2010). Frequency distribution of antimalarial drug resistance alleles among plasmodium falciparum isolates from Gezira State, Central Sudan, and Gedarif State, Eastern Sudan. Am. J. Trop. Med. Hygiene.

[16] Bashey F (2015). Within-host competitive interactions as a mechanism for the maintenance of parasite diversity. Philos. Trans. R. Soc. B Biol. Sci..

[17] Leemans I, Hooshmand-Rad P, Uggla A (1997). The indirect fluorescent antibody test based on schizont antigen for study of the sheep parasite *Theileria lestoquardi*. Vet. Parasitol..

[18] Goh S, Ngugi D, Lizundia R, Hostettler I, Woods K, Ballingall K, MacHugh ND, Morrison WI, Weir W, Shiels B (2016). Identification of Theileria lestoquardi antigens recognized by CD8+ T cells. PLoS ONE.

[19] Morrison WI (2009). Progress towards understanding the immunobiology of Theileria parasites. Parasitology.

[20] Leemans I, Brown D, Hooshmand-Rad P, Kirvar E, Uggla A (1999). Infectivity and cross-immunity studies of *Theileria lestoquardi* and *Theileria annulata* in sheep and cattle: I. In vivo responses. Veterinary Parasitol..

[21] Pacheco MA, Ryan EM, Poe AC, Basco L, Udhayakumar V, Collins WE, Escalante AA (2010). Evidence for negative selection on the gene encoding rhoptry-associated protein 1 (RAP-1) in Plasmodium spp. Infect. Genet. Evol..

[22] Mideo N (2009). Parasite adaptations to within-host competition. Trends Parasitol..

[23] Bruce MC, Donnelly CA, Alpers MP, Galinski MR, Barnwell JW, Walliker D, Day KP (2000). Cross-species interactions between malaria parasites in humans. Science.

[24] Morrison W, McKeever D (2006). Current status of vaccine development against Theileria parasites. Parasitology.

[25] Awad H, Al-Hamidhi S, El Hussein A-RM, zein Yousif YM, Taha KM, Salih DA, Weir W, Babiker HA (2018). *Theileria lestoquardi* in Sudan is highly diverse and genetically distinct from that in Oman. Infect. Genet. Evol..

[26] Smith T, Felger I, Kitua A, Tanner M, Beck H-P (1999). Dynamics of multiple Plasmodium falciparum infections in infants in a highly endemic area of Tanzania. Trans. R. Soc. Trop. Med. Hygiene.

[27] Staroscik, A., dsDNA copy number calculator*. URI Genomics & Sequencing Center*, (2004).

[28] Burnham KP, Anderson DR (2003). Model Selection and Multimodel Inference: A Practical Information-Theoretic Approach.

