# Supplementary material for: Dynamics and within-host interaction of Theileria lestoquardi and T. ovis among naive sheep in Oman
Source: Sci Rep. 2020 Nov 13;10:19802. doi: 10.1038/s41598-020-76844-2 (PMC7666211; doi:10.1038/s41598-020-76844-2)
Supplement: Supplementary file 2 — Supplementary Legend. [file 41598_2020_76844_MOESM2_ESM.docx]

**Supplementary table 1.** Density of *T. lestoquardi* and *T. ovis* and microsatellites of *T. lestoquardi* among the examined sheep
